# Risk Assessment and Determination of Factors That Cause the Development of Hyperinsulinemia in School-Age Adolescents

**DOI:** 10.3390/medicina58010009

**Published:** 2021-12-22

**Authors:** Igor Lukic, Nikola Savic, Maja Simic, Nevena Rankovic, Dragica Rankovic, Ljubomir Lazic

**Affiliations:** 1 Faculty of Medical Sciences, University of Kragujevac, 34000 Kragujevac, Serbia; igorlukicvaljevo@gmail.com (I.L.); nikolasavicvzs@gmail.com (N.S.); 2 Clinic for Aesthetic Surgery Ribnikar, 11000 Belgrade, Serbia; maja.lazic87@gmail.com; 3 Department of Computer Science, School of Computing, Union University, 11000 Belgrade, Serbia; 4 Department of Mathematics and Statistics, Faculty of Applied Sciences in Nis, Union University “Nikola Tesla”, 18000 Nis, Serbia; nr17031994@gmail.com; 5 Department of Computer Engineering, School of Computing, Union University, 11000 Belgrade, Serbia; ljlazic@raf.rs

**Keywords:** hyperinsulinemia, risk factors, adolescents, BMI, ANN

## Abstract

*Background and Objectives*: Hyperinsulinemia and insulin resistance are not synonymous; if the risk of developing insulin resistance in adolescents is monitored, they do not necessarily have hyperinsulinemia. It is considered a condition of pre-diabetes and represents a condition of increased risk of developing DM (diabetes mellitus); it can exist for many years without people having the appropriate symptoms. This study aims to determine the risk of developing hyperinsulinemia at an early age in adolescents by examining which factors are crucial for its occurrence. *Materials and Methods*: The cross-sectional study lasting from 2019 to 2021 (2 years) was realized at the school children’s department in the Valjevo Health Center, which included a total of 822 respondents (392 male and 430 female) children and adolescents aged 12 to 17. All respondents underwent a regular, systematic examination scheduled for school children. BMI is a criterion according to which respondents are divided into three groups. *Results*: After summary analyzes of OGTT test respondents and calculated values of *HOMA-IR* (homeostatic model assessment for insulin resistance), the study showed that a large percentage of respondents, a total of 12.7%, are at risk for hyperinsulinemia. The research described in this paper aimed to use the most popular AI (artificial intelligence) model, ANN (artificial neural network), to show that 13.1% of adolescents are at risk, i.e., the risk is higher by 0.4%, which was shown by statistical tests as a significant difference. *Conclusions*: It is estimated that a model using three different *ANN architectures*, based on Taguchi’s orthogonal vector plans, gives more precise and accurate results with much less error. In addition to monitoring changes in each individual’s risk, the risk assessment of the entire monitored group is updated without having to analyze all data.

## 1. Introduction

The primary source of energy for every organism is glucose. When the β cells of the pancreas secrete the hormone insulin, to a greater extent than necessary, it begins to stick to the cell membrane and does not allow glucose to enter the body’s cells, but it remains in the blood. Then, laboratory blood tests can determine that both insulin and glucose values or only insulin have increased. The pancreas stimulates insulin secretion to allow glucose to enter cells [1,2]. The causes of such metabolic changes are different. However, as a result, there are increased concentrations of both insulin and glucose, which can cause many unwanted consequences, primarily elevated insulin levels known as hyperinsulinemia, which, over time, contributes to a high risk of developing type 2 diabetes and many heart diseases. Insulin resistance (IR) is considered a condition in which the cells of the body do not respond to the action of insulin, so that insulin cannot perform its function. Insulin remains present in higher concentrations, and in order to counteract cell resistance, the organism produces even more significant amounts of insulin, its level in the blood increases, and hyperinsulinemia occurs [3,4,5].

A pre-diabetic condition occurs if the values of insulin and or insulin and glucose are increased, which results in an increased risk of developing macrovascular diseases and neuropathy. About 30% of such individuals develop type 2 DM within ten years [6]. Risk factors vary, starting from genetic predispositions, metabolic syndrome, polycystic ovaries, obesity, various hormonal disorders, insufficient physical activity, long-term consumption of certain drugs, alcohol, or other harmful substances [7,8]. In addition to the above factors, stress and improper diet significantly contribute to this disorder in the body. All the above indicate that the only way to prevent or mitigate the development of such a gradual disease is early diagnosis and timely elimination of the cause of its occurrence [9]. That is why it is essential to start at the earliest age with monitoring and discovering possible risk factors that could be eliminated promptly [10]. This is possible through regular, systematic examinations of children and adolescents in an outpatient setting by monitoring basic laboratory parameters such as BMI, measuring blood pressure, cholesterol, glucose, and other laboratory tests, but also by monitoring other factors that have a significant influence, such as hereditary factors, socio-economic, nutrition, mental health, stress, physical activity, etc. The study that will be presented in this paper aims to show the degree of risk in which children and adolescents are and which factors are most likely to lead to hyperinsulinemia [1,4,11].

The research aims to compare the results obtained by statistical calculations and a model that uses different architectures of ANN depending on the number of risk factors. Today, there are more modern and reliable ways of risk assessment, such as artificial intelligence tools, that will be presented in this paper. Each ANN architecture is constructed based on Taguchi’s orthogonal vector plans. The new proposed methodology aims to more quickly and efficiently show all the changes in assessing the risk of hyperinsulinemia in adolescents:

1. The risk assessment of each adolescent may suffer.
2. Changes in the entire study group, i.e., population, can be monitored because each change is updated without re-analyzing all data.
3. The risk can be monitored for a certain period.
4. Risk assessment can be monitored according to set criteria, without additional analysis.
5. Risk assessment is more efficient with minor model errors.

## 2. Related Work

An increasing number of studies conducted in our country indicate that young people in Serbia have a high frequency of various factors that significantly affect adolescents’ health and proper development [8]. Additionally, research findings indicate that, in addition to improper diet and obesity with reduced physical activity, metabolic syndrome results in hyperinsulinemia [12] for insulin resistance [8]. Several studies have shown that insulin resistance increases with the onset of puberty, reaches a maximum in the third stage, according to Tanner [13], and returns to prepubertal levels at the end of puberty. One study showed a 30% reduction in insulin sensitivity during puberty [14,15]. These changes in insulin values are assumed to be due to the anabolic effects of growth hormone and insulin during accelerated growth and development at puberty [16]. The results of previous research indicated that the change in insulin sensitivity is a consequence of a change in fat distribution, while newer research indicates the same degree of reduction in insulin sensitivity in both lean and obese children [17]. One of the most important components, hyperinsulinemia, is one of the criteria for developing type 2 diabetes, according to the International Diabetes Federation [18]. Hyperinsulinemia is the pathophysiological basis for numerous metabolic and non-metabolic disorders. It represents a significant risk factor for cardiovascular diseases in adolescence, and if preventive measures are not taken in the youth population, an increase in morbidity and mortality can be expected in the next generations of adults. Numerous studies have shown that proper nutrition, greater physical activity, changes in quality, and lifestyle can reduce disease risk [19,20]. All preventive activities for these reasons must be carried out from the earliest age. It is essential to introduce adolescents to the disease and the consequences that it can cause [21]. An accurate expert assessment of the risk of its occurrence is also necessary [22]. The most important step in preventing any disease, including hyperinsulinemia, is preventive action to reduce the risk and mitigate it as much as possible. It is important for each individual but also for society as a whole [23]. Hyperinsulinemia is diagnosed using the oral glucose tolerance test, the glucose tolerance test (OGTT), and based on the values of the insulin sensitivity index [24,25,26,27]. Artificial neural networks, as one of the most powerful tools of artificial intelligence, are increasingly used in medicine. They play a unique role in medical diagnostics, dentistry, internal medicine, and other fields. Due to the possibility of modeling various complex systems, which have many conditional, indeterminate, and unknown factors, ANN is expected to be increasingly used in the future [28,29].

## 3. Research Study

### 3.1. Sample Structure

A total of 822 respondents, adolescents from primary and secondary schools from the territory of six municipalities of the Kolubara district, aged 12 to 17, participated in the research that will be presented, who applied for a regular, systematic examination. Before the beginning of the study, the approval of the ethics board of the Health Center in Valjevo was obtained. After their approval, before the start of the research, the respondents, in our case, their parents, were acquainted with the purpose and procedure of the research. The sample consists of 392 (47.7%) male respondents and 430 (52.3%) female respondents. The environment from which the respondents came included urban area 318 (38.7%), suburban area 272 (33.1%), and rural area 232 (28.2%), and the average age was 14.5 ± 2.1. The study group consisted of 102 (12.4%) respondents who had *HOMA-IR* values greater than 1, calculated after the OGTT test, and were at risk compared to the control group 85 (10.3%), which consisted of respondents with average OGTT test values and *HOMA-IR* values approximately equal to 1, i.e., they were not at risk.

### 3.2. Subject and Goal of the Research

The subject of research is to determine the risk of developing hyperinsulinemia in adolescents. The research also aims to determine the influence of various risk factors such as obesity, improper diet, hereditary diseases, and others. The research is presented by various statistical analyses and concluding remarks and was performed from 2019 to 2021 (2 years). It was realized in two phases: first, data were collected during the systematic examination of students, and second, after additional analyses performed on a pediatrician’s recommendation. In the first phase, upon arrival for a systematic examination in the presence of parents or teachers, adolescents filled out a questionnaire with general information such as gender, the environment they come from, assessment of their general health, socioeconomic living, and learning conditions. The second part of the question was related to eating and consuming certain foods, drinks, dieting, smoking habits, alcohol, or taking psychoactive substances. In the third part of the questionnaire, adolescents stated the degree of physical activity and its impact on health [18,19]. Specialist doctors performed the second part of the examination. Adolescents were then measured for weight, height, waist circumference, blood pressure, experienced stress, family history, and basic laboratory analyses of glucose, erythrocytes, platelets, lymphocytes, HDL, LDL, triglycerides, sedimentation, and CRP (C reactive protein). Respondents, based on the first part of the examination, if necessary, referred to the second part of the examination to do the OGTT test [1,30,31,32,33].

### 3.3. Applied Methodology

Factor analysis will group the factors and examine the impact on the risk of developing hyperinsulinemia: exploratory and confirmatory. The Kruskal–Wallis, ANOVA, and Chi-square (χ^2^) tests will also be used to determine the relationship between individual factors. Moreover, the Pearson and Spearman correlation coefficients will be used to determine the degree of correlation between individual independent variables. Statistical significance will be adopted at a probability level of *p* < 0.05. If the glucose values after 120 min, after the end of the test, are higher than 11.1 mmol/L, type 2 diabetes is diagnosed. If the glucose values after the end of the test in 120 min are between 7.7 mmol/L and 11.1 mmol/L, the diagnosis of glucose intolerance is made. Furthermore, if glucose values return below 7.7 mmol/L to reference values, then glucose tolerance is standard. Initial insulin should be less than 10 uIU/mL at 0 min, preferably less than 6 uIU/mL. After 120 min of glucose ingestion, insulin levels should be as close to baseline as possible and less than 24.9 uIU/mL if the values are higher than baseline. The criteria for monitoring the risk are the values of the OGTT test performed based on body mass index (BMI). All respondents, 187 (22.7) with BMI greater than 24, were divided into three groups I group: BMI (24 to 27), group II BMI (27 to 29), and the third group BMI (greater than 29). These three groups represented the study group, while the remaining 85 (10.4%) were the control group, where the respondents with the OGTT test had values that met the predicted reference values for each parameter. *HOMA-IR* is an index of insulin sensitivity, calculated from pairs of insulin and glycemia, according to Formula (1):

(1) $$\;\;\;\;\;\;\;\;\;\;\;\;\;\;\;\;\;\;HOMA-IR=\frac{Insulin\;in\;0\;min.\;\;\times \;Glucose\;in\;0\;min.}{22.5}\;$$

*HOMA-IR* in healthy individuals has a value of 1. Values from 1 to 1.6 represent peripheral insulin resistance, while values from 1.6 to 2 are defined as prehyperinsulinemia. *HOMA-IR* values greater than two were defined as hyperinsulinemia [33,34]. If the insulin values are elevated, the diagnosis of insulin resistance, hyperinsulinemia, or even type 2 diabetes is made based on glucose [35,36].

#### 3.3.1. Statistical and Factor Analysis

The following were used in the statistical analysis of the data: Kruskal–Wallis H test, Pearson’s χ^2^ test, and paired samples test (*t*-test). Reliable tests were used to analyze the differences of discrete variables. In order to determine the factors that have the most dominant influence, factor analysis was used. Principal component analysis (PCI) reduces a more extensive set of variables to a smaller number of components that affect the maximum number of variants of a set of variables. Exploratory factor analysis (EFA) defines a set of research variables to obtain the most reliable solution. Confirmatory factor analysis (CFA) was used to test the hypotheses of a given model if there is a connection between the selected factors and the variables that are analyzed in this way. Correlations between continuous variables were analyzed by the Kruskal–Wallis H test and the paired samples test. Probabilities: less than 0.05 are considered significant statistical differences. Mean values with standard deviation (±SD) were calculated.

#### 3.3.2. Artificial Neural Networks

Artificial neural networks, as a powerful tool of artificial intelligence, are increasingly used in medicine. They form a system consisting of nodes or neurons interconnected by connections, through which data are transmitted. The architecture of each network consists of three parts: the input layer, the hidden layer, and the output layer. The input layer can have multiple input sizes, through which data are received. There can be one or more hidden layers, and they are used for data processing according to a given criterion, depending on the problem being solved. The output layer may have one or more output values. The strength of the connection between neurons is called the weighting factor. First, it is necessary to train the neural network and train for its further use. The goal of our proposed model is to select the most straightforward neural network architecture, with as few iterations as possible and shortening training, testing, and validation times. The main idea is to use a robust experiment design method based on Taguchi’s orthogonal vector plans [35,36] (See Appendix A).

**Figure 1:**
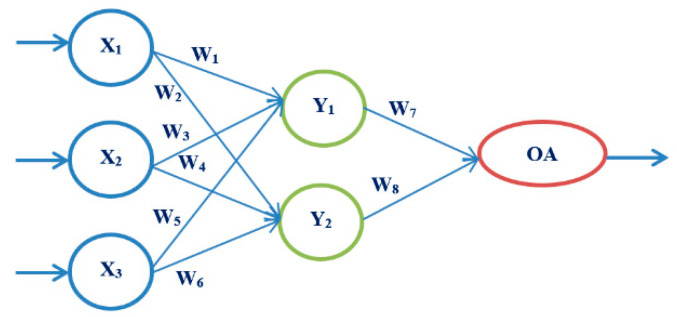
ANN architecture with one hidden layer based on OA18 (ANN-OA18).

**Figure 2:**
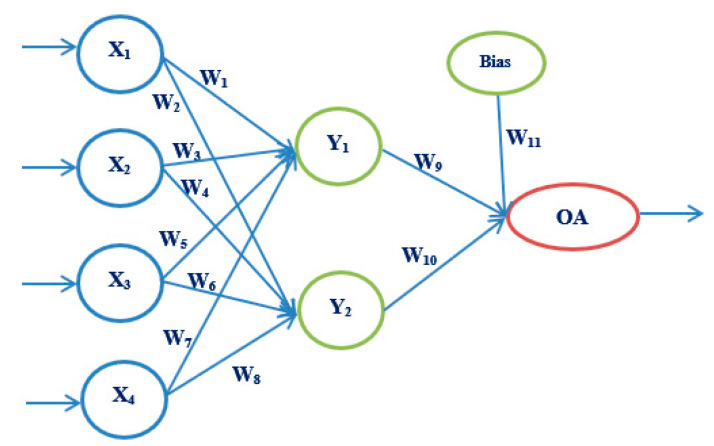
ANN architecture with one hidden layer based on OA12 (ANN-OA12).

**Figure 3:**
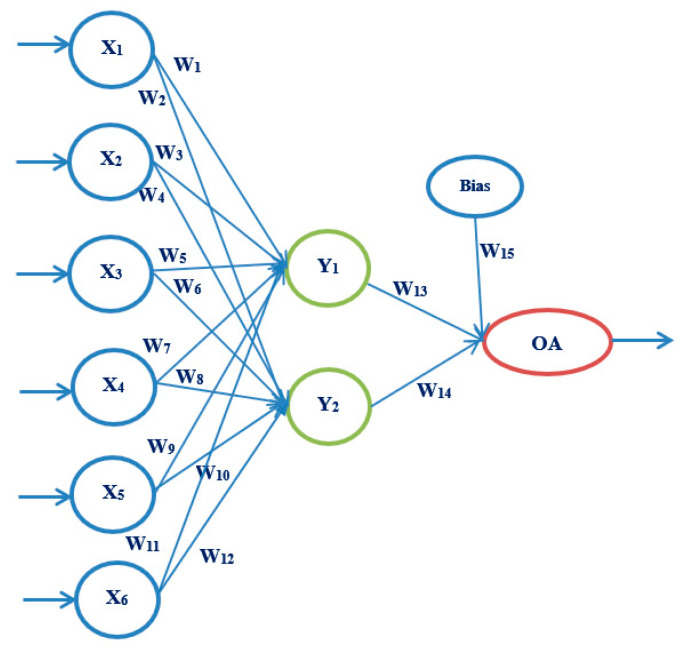
ANN architecture with one hidden layer based on OA16 (ANN-OA16).

The following artificial neural network architectures were used: ANN-OA18 based on Taguchi’s vector plan OA18 with three input values, ANN-OA12 based on Taguchi’s vector plan OA12 with four input quantities, and ANN-OA16 based on Taguchi’s vector plan OA16 with six input values Figure 1, Figure 2 and Figure 3.

## 4. Analysis of the Obtained Results

The average age of the respondents was 14.5 ± 2.1. Weight (66.0 ± 8.1), height (165.8 ± 7.3), waist circumference (69.3 ± 7.4), and BMI (24.1 ± 3.4). The analysis of the obtained results revealed that 187 (22.7%) respondents had symptoms that could indicate a risk of developing hyperinsulinemia. This group of respondents was referred for additional examinations by a pediatrician. Respondents with high values of either glucose or insulin or both parameters formed the study group; the remaining respondents with the OGTT test and had glucose and insulin values within average values formed the control group.

### 4.1. Results of Factor Analysis

The exploratory factor analysis singled out four factors that have the most significant influence on the risk of developing hyperinsulinemia. These are BMI with a variance of 23.28%, cholesterol with a variance of 15.80%, hereditary factor with a variance of 13.71%, and physical activity with a variance of 11.17%. Confirmatory factor analysis established that BMI is the most influential factor with a variance of 49.57%.

The obtained results were classified and analyzed according to the BMI value. The first group consists of 107 (13.0%) respondents, with BMI values in the range of 24 kg/cm^2^ to 27 kg/cm^2^, the second group consists of respondents with BMI values in the range of 27 kg/cm^2^ to 29 kg/cm^2^, and the third group with values BMI greater than 29 kg/cm^2^. Mean BMI for group I is (25.4 ± 0.8), for group II is (27.8 ± 0.5), and for group III is (30.6 ± 1.2). It can be concluded that, using the χ^2^ test, there are statistically significant differences in the obtained BMI values at the level of all three groups (χ^2^ = 96.062, *p* = 0.000). Analyzing the values within each group, the Kruskal–Wallis test showed that there were no statistically significant differences for the first group (KW (H) = 0.334, *p* = 0.563), for the second group (KW (H) = 0.541, *p* = 0.462), and third group (KW (H) = 0.461, *p* = 0.497).

### 4.2. OGTT and HOMA-IR Test Results

The results of the OGTT test, performed by the respondents on the recommendation of a pediatrician show that, in all three groups of respondents, there were no significant differences in the values of both glucose and insulin in 0 min. Glucose in 0 min for group I is (4.7 ± 0.6), for group II is (4.9 ± 0.4), and group III is (5.2 ± 0.4). Insulin in 0 min for group I is (16.7 ± 4.3), for group II is (25.7 ± 3.3), and group III is (31.4 ± 3.5). Glucose for the first group is (KW (H) = 0.001, *p* = 0.970), for the second group is (KW (H) = 0.004, *p* = 0.947), and the third group is (KW (H) = 0.715, *p* = 0.398). Insulin for the first group is (KW (H) = 0.006, *p* = 0.940), for the second group is (KW (H) = 1.231, *p* = 0.267), and the third group is (KW (H) = 0.126, *p* = 0.722).

At 60 min, there are significant differences in insulin, while there is no difference in glucose. Glucose in 60 min for group I is (6.4 ± 0.7), for group II is (7.2 ± 0.6), and group III is (8.0 ± 0.6). Insulin in 60 min for group I is (66.2 ± 22.4), for group II is (78.4 ± 22.7), and group III is (90.6 ± 13.8). Glucose for the first group is (KW (H) = 0.210, *p* = 0.647), for the second group is (KW (H) = 0.179, *p* = 0.672), and the third group is (KW (H) = 0.463, *p* = 0.496). Insulin for the first group is (KW (H) = 56.45, *p* = 0.000), for the second group is (KW (H) = 24.033, *p* = 0.000), and the third group is (KW (H) = 6.632, *p* = 0.010).

Insulin and glucose values in the 120 min OGTT test are different; some are significant differences, and some are not. Glucose in 120 min for group I is (5.0 ± 0.6), for group II is (5.5 ± 0.4), and group III is (6.3 ± 0.9). Insulin in 120 min for group I is (28.7 ± 25.8), for group II is (39.3 ± 11.8), and group III is (53.4 ± 16.6). Glucose for the first group is (KW (H) = 0.137, *p* = 0.711), for the second group is (KW (H) = 3.346, *p* = 0.067), and for the third group, there are significant differences, with (KW (H) = 5.130, *p* = 0.024). Insulin in the first group has significant differences, with (KW (H) = 4.937, *p* = 0.026); in the second group, there are significant differences, with (KW (H) = 4.494, *p* = 0.034); and the third group has no differences (KW (H) = 0.176, *p* = 0.675). 

Based on the obtained OGTT test values, the *HOMA-IR* value was calculated for all values. Out of a total of 187 (22.7%) respondents, 44 (5.3%) had 1.0–1.6 values, of which 19 (2.3%) were male and 25 (3.0%) were female respondents, and they were considered to be at low risk for developing hyperinsulinemia. *HOMA-IR* 1.6–2 values were present in 42 (5.1%) respondents, 15 (1.8%) males, and 27 (3.3%) females, and they were at medium risk of prehyperinsulimia. *HOMA-IR* values > 2 were present in 16 (1.9%) respondents, 5 (0.6%) male and 11 (1.3%) female respondents, and the presence of hyperinsulinemia could be diagnosed.

**Figure 4:**
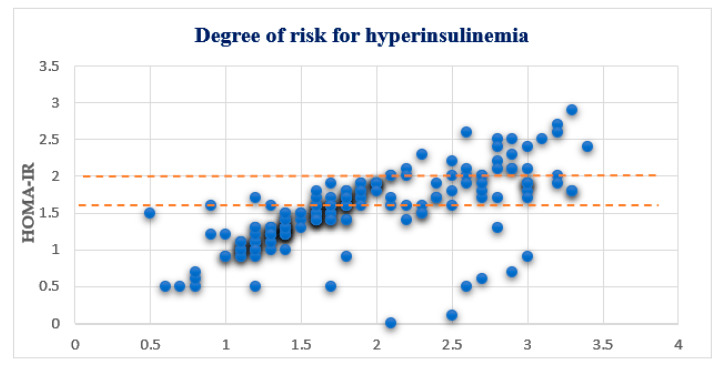
Degree of risk for hyperinsulinemia.

**Figure 5:**
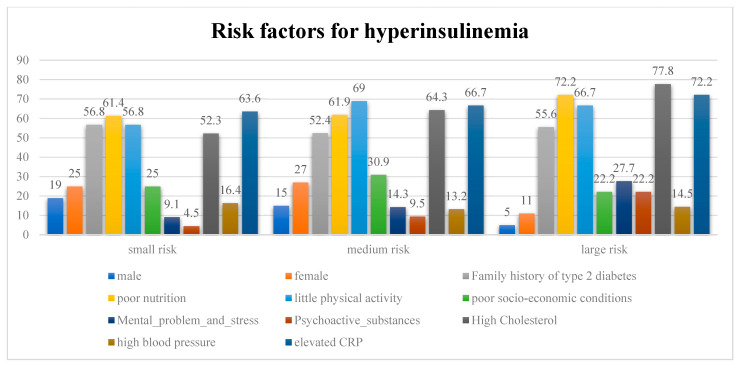
Risk factors for hyperinsulinemia.

The degree of risk for hyperinsulinemia is given in Figure 4 while the risk factors are shown in Figure 5.

### 4.3. Risk Factors

Differences in the risk of developing hyperinsulinemia also exist by gender in each group, as shown by the Kruskal–Wallis test (KW (H) = 5.824, *p* = 0.016). By checking the family history of the respondents, it can be determined that there are no statistically significant differences between the respondents in all three study groups (KW (H) = 1.305, *p* = 0.253), but that the percentage of respondents with a positive family history is very high: 56.8% in group I, 52.4% in group II, and 62.5% in group III. When it comes to improper diet, there are significant differences in the respondents (KW (H) = 4.105, *p* = 0.046). The percentage of respondents who eat improperly is extremely high in group I (54.7%), in group II (61.9%), and group III (81.3%). Poor physical activity was suppressed in a large percentage of respondents, but there were no significant differences between them (KW (H) = 0.698, *p* = 0.403). Thus, in group I, this was 56.8%; in group II, 69.0%; and group III, 66.7%. There are no differences among respondents when it comes to socio-economic conditions (KW (H) = 3.328, *p* = 0.068). Between one-third and one-quarter of them have poor conditions: in group I, 25.0%, in group II, 30.9%, and group III, 22.2%. Mental problems and stress are present to a lesser extent in all three groups, and there are significant differences (KW (H) = 0.068, *p* = 0.794). In group I, these problems are present in 9.1% of respondents; in group II, 14.3%; and in group III, 18.7%. There are no significant differences when it comes to the use of various psychoactive substances (PAS); the largest percentage of respondents use alcohol and cigarettes (KW (H) = 3.508, *p* = 0.061). In group I, this was 4.5%; in group II, 9.5%; and in group III, 6.3%, which is less than 10% in all groups. By measuring cholesterol, it was found that there are significant differences (KW (H) = 4.459, *p* = 0.006). In group I, 52.3% of the respondents had elevated cholesterol; in group II, this was 64.3%, and in group III, this was 75.0%. Significant differences also exist in the respondents when elevated blood pressure is observed (KW (H) = 5.333, *p* = 0.021). In group I, 16.4% respondents had high blood pressure; in group II, 13.2% did; and in group III, it was 43.7%. Increased CRP is present in a high percentage in all three groups of respondents, and there are significant differences between them (KW (H) = 11.466, *p* = 0.001). There were 63.6% in group I, 66.7% in group II, and 81.3% in group III. The values of total protein were measured: in group I, this was (79.3 ± 3.1) g/L in 16 (36.4%) respondents; in group II, it was (85.6 ± 4.5) g/L in 19 (45.2%) respondents, and in group III, it was (94.7 ± 5.1) g/L in 8 (50.0%) respondents. HDL values were measured: in group I, this was (1.4 ± 0.3) mmol/L in 13 (29.5%) respondents; in group II, it was (1.1 ± 0.5) mmol/L in 16 (38.1%) respondents; and in group III, it was (0.8 ± 0.4) mmol/L in 10 (62.5%) respondents. LDL values were measured: in group I, this was (4.1 ± 2.4) mmol/L in 15 (34.1%) respondents; in group II, it was (4.4 ± 0.3) mmol/L in 19 (45.2%) respondents; and in III group, it was (4.7 ± 0.5) mmol/L in 9 (56.3%) respondents. Triglyceride values were measured: in group I, this was (3.1 ± 1.5) mmol/L in 16 (36.4%) respondents; in group II, it was (4.8 ± 0.9) mmol/L in 17 (40.5%) respondents; and in group III, it was (5.8 ± 1.7) mmol/L in 10 (62.5%) respondents.

A total of 102 (12.3%) in the study group are at risk: 5.4% of the sample is at lower risk of insulin resistance, 5.1% is at medium risk of prehyperinsulinemia, and 1.9% of respondents can be diagnosed with hyperinsulinemia.

Out of a total of 187 (22.7%) respondents sent to have the OGTT test, 85 (10.4%) were not at risk, with glucose and insulin values within average values.

### 4.4. Results Obtained Using ANN

**Figure 6:**
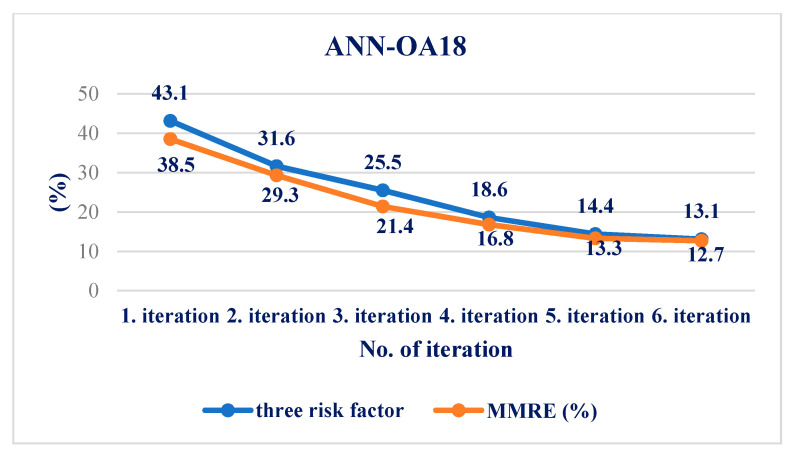
Influence of three factors on the resulting risk and error of the ANN-OA18 model through six iterations.

**Figure 7:**
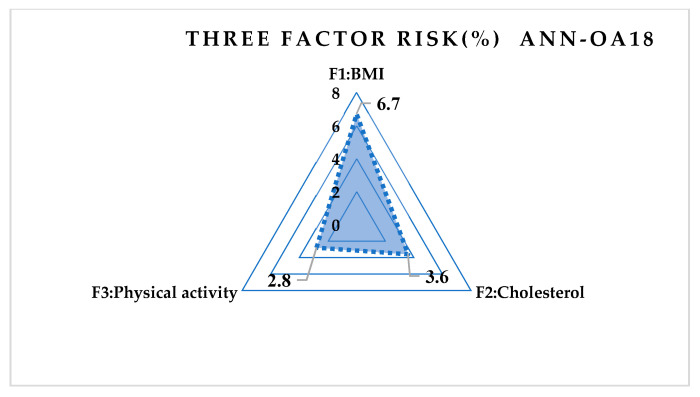
Influence of three risk factors on ANN-OA18.

For respondents with three risk factors, the ANN-OA18 architecture was used, based on Taguchi’s combined orthogonal vector plan OA18, which has three input sizes of eight weighting coefficients and two levels (OA18 = 2^1^ 3^7^), one hidden layer with two nodes, and one output value. As shown in Figure 1 samples with at least three risk factors were taken, which means that respondents with more than three factors were included. The GA criterion was met after six iterations; the risk was 13.1% compared to the sample, with an error of 12.7% Figure 6 and Figure 7.

**Figure 8:**
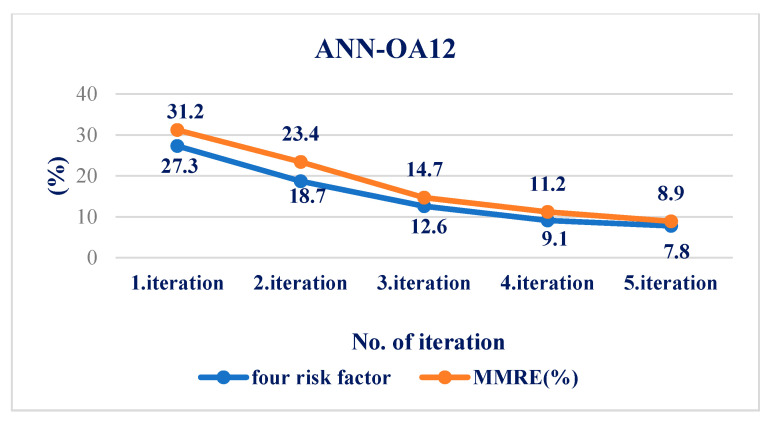
Influence of four factors on the resulting risk and error of the ANN-OA12 model through five iterations.

**Figure 9:**
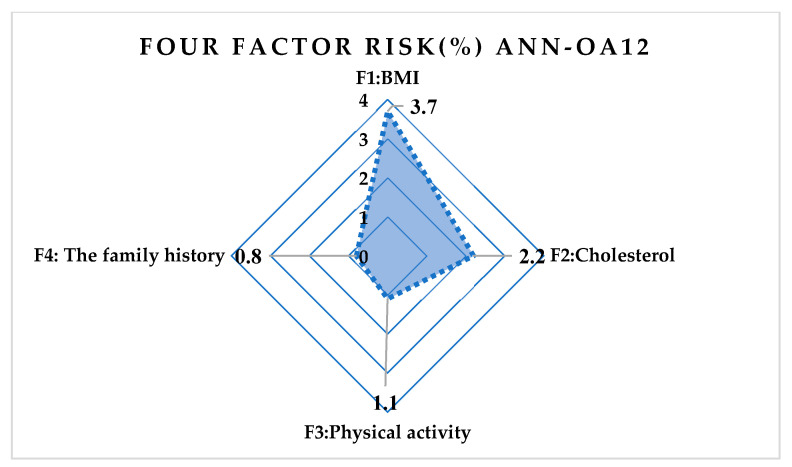
Influence of four risk factors on ANN-OA12.

For respondents with four risk factors, the ANN-OA12 architecture was used, based on Taguchi’s orthogonal vector plan OA12, which has four input quantities, eleven weighting coefficients and one level (OA12 = 2^11^), and one hidden layer with two nodes and one output value Figure 2. Samples with at least four risk factors were taken, which means that respondents with more than four factors were included. GA criterion is met after five iterations, and the risk is 7.8% compared to the sample, with an error of 8.9% Figure 8 and Figure 9.

**Figure 10:**
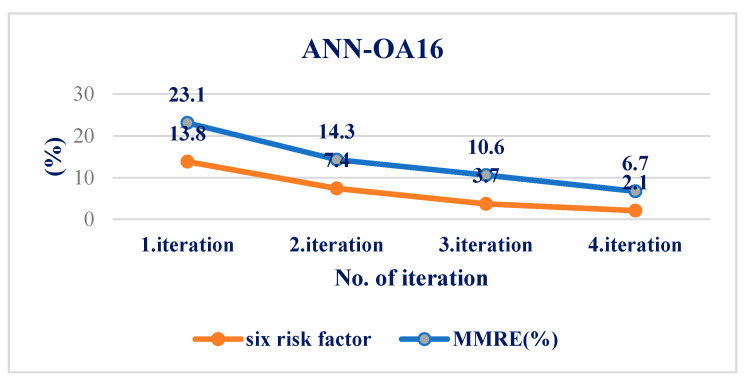
Influence of six factors on the resulting risk and error of the ANN-OA16 model through four iterations.

**Figure 11:**
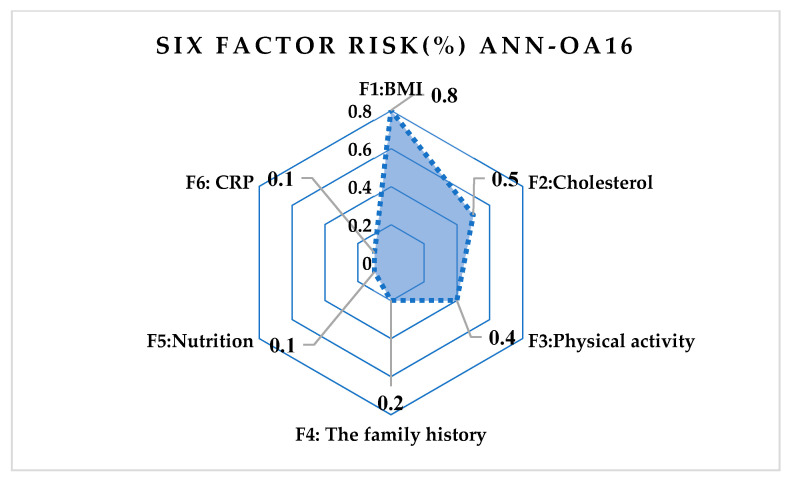
Influence of six risk factors on ANN-OA16.

For respondents with six risk factors, the ANN-OA16 architecture was used, based on Taguchi’s orthogonal vector plan OA16, which has six input sizes of fifteen weighting coefficients and one level (OA16 = 2^15^) of a hidden layer with two nodes and one output value. As shown in Figure 3, samples with at least six risk factors were taken, which means that respondents with more than six factors were included. GA criterion is met after four iterations, and the risk is 2.1% compared to the sample, with an error of 6.7% Figure 10 and Figure 11.

The correlation between the estimated and real values for all three architectures, ANN-OA18, ANN-OA12, and ANN-OA16, are given in Table 1. We conclude that the ANN-OA16 architecture has the highest values of all correlation coefficients and the smallest number of iterations for which it meets the GA criterion.

## 5. Discussion

The study aimed to determine the risk of developing hyperinsulinemia in adolescents and the factors that influence its occurrence. The analysis of the obtained results shows that female respondents are at higher risk than male respondents in the first group after the results of the OGTT test, where *HOMA-IR* was calculated, and less than 1.6 (5.3%) of the respondents were at low risk of developing hyperinsulinemia. The number of female respondents is 3.0% compared to males, which is 2.3% of the whole sample. In the second group, 5.1% are at medium risk, of which 3.3 are female respondents, almost twice as many as the 1.8% of male respondents. In the third group, where hyperinsulinemia can be diagnosed, 3.8% are female respondents, and 1.6% are male respondents. Another check using the paired samples test (*t*-test) for comparative analysis of glucose and insulin before and after the OGTT test showed statistically significant differences in the values of both glucose and insulin, which means that there is a risk. The influence of hereditary factors is in a large percentage, but it is present in all groups. There are significant differences in improper diet and poor physical activity among the respondents. Socio-economic conditions do not have a significant impact on the risk of disease. Psychological problems, stress, and psychoactive substances are also in a smaller percentage, and there is no difference between the respondents in all three groups. Significant differences exist in respondents who have elevated cholesterol, blood pressure, and CRP. The third group has 75.0% of respondents with elevated cholesterol, 43.7% with elevated blood pressure, and 81.3% with increased CRP values.

Using a comparative model of artificial intelligence with three different *ANN architectures*, the results obtained are much more accurate. The risk error in group I was 25.8%, while in the same group, the error in ANN-OA18 was 12.7%. In group II, the statistical error is 11.8%, while in the same group, with ANN-OA12, the error is 8.9%. The error was 16.6% in group III, while in the same group, with ANN-OA16, the error was 6.7%. Our study showed that 38.2% of male respondents were at risk of hyperinsulinemia in the study group, and a significantly higher number of female respondents was 61.7%. A similar study with adolescents was conducted in Portugal [33], where the total number at risk was about 72% of adolescents. In a study conducted at Santa Catarina State University in Brazil [32], 52.9% of male respondents and 67.7% of female respondents were at risk.

The advantage of non-parametric models concerning classical statistical non-parametric models is the speed and accuracy of determining the required output values, shown by the results in this paper. For any change in the input values, three different *ANN architectures* can immediately assess the risk of developing hyperinsulinemia.

## 6. Conclusions

The presented research was analyzed through two approaches, statistical tests and the use of ANN. Both approaches have shown that adolescents are at low, medium, or high risk of developing hyperinsulinemia. Analyzing statistical tests, 12.7% of adolescents were at risk. Using three different *ANN architectures*, 13.1% of adolescents were at risk, and it was shown that there were statistically significant differences, which was the aim of this study. Analyzing all the factors that could lead to hyperinsulinemia, it was determined that female respondents are at higher risk than male respondents, and that a hereditary factor is present in a high percentage in all groups. The most significant risk is improper diet and reduced physical activity, which leads to obesity and increased BMI, especially in puberty.

As a consequence of this condition, the respondents have a high percentage of elevated cholesterol, a medium percentage of elevated blood pressure, and a very high percentage of increased CRP. Factor exploratory analysis identified BMI as the most influential risk factor and participated with 49.6% in the total risk. In addition, the confirmatory analysis identified three other significant risk factors: high cholesterol, family heredity, and poor physical activity.

Since the research began before the onset of the COVID-19 pandemic, until the third wave when a small percentage of children and adolescents became ill, this research did not consider the impact of the virus as one of the factors. Subsequent research aims to examine adolescents who have contracted the COVID-19 virus and its impact on the development of hyperinsulinemia.

## Figures and Tables

**Table 1:** Correlation coefficients—Pearson and Spearman—and R^2^ values using different kernel functions.

Correlation	ANN-OA18	ANN-OA12	ANN-OA16
Pearson	0.743	0.853	0.872
Spearman	0.726	0.845	0.925
R^2^ Linear	0.815	0.827	0.934
R^2^ Quadratic	0.826	0.868	0.958
R^2^ Cubic	0.967	0.893	0.875
MMRE (%)	12.7	8.9	2.1

## Data Availability

The data is currently not available because it will be a part of Ph.D. thesis of author Igor Lukic.

## Appendix A

Algorithm—Taguchi robust design of the experiment.

**Step 1:** Each of the three architectures used is a simple artificial neural network with a single input layer. In the experiment presented, the input layer consists of three input risk factors for ANN-OA18, four input risk factors for ANN-OA12, and six risk factors for ANN-OA16.

**Step 2:** The values of all examined factors are represented by different values and in different units of measurement. That is why it is necessary to translate them into uniform values. In this way, all factors are equally represented and have the same impact on the risk of hyperinsulinemia. All input values are transformed according to the following formula: The function µD (X): R → [0, 1], translates the real values of input values into coded values from the interval [0, 1], in the following way (A1): [35,36].

µD (Y_i_) = (X_i_ − X_min_)/(X_max_ − X_min_) (A1)

where D is the set of data on which the experiment is performed, X_i_ is the input value, X_min_ is the smallest input value, and X_max_ the greatest input value on the observed dataset [3].

**Step 3:** The sigmoid function as the activation function of the hidden layer was used (A2):

(A2) $$Y_{i}=\frac{1}{1+e^{-X_{i}}}\;i=\bar{1,n}$$

In the first proposed ANN-OA18 architecture, an orthogonal plan of two and three levels L1, L2, and L3 and the initial values of the weighting factors W*_i_* that take the values from the interval [−1, 1] and [−1, 0, 1] were used. The second proposed architecture, ANN-OA12, and the third architecture, ANN-OA16, has an orthogonal plan of three levels L1, L2, and L3, and the initial values of the weighting factors Wi that take the values from the interval [−1, 1]. For each subsequent iteration, new weight factor values must be calculated as follows (e.g., for ANN-OA12 architecture) [1,2]:

**Step 4:** Method of defuzzification was used according to Formulas (A3) and (A4):

*Y_i_* = (*X_min_* + *µ**D* (*X_i_*))…(*X_max_ − X_min_*) (A3)

(A4) $$Risk\left(i\right)=OA\left(ANN_{i}\right)=\frac{1}{n}\sum_{i=1}^{n}Y_{i},\;\mathrm{where}\;i=18,\;i=12,\;i=16$$

where *OA*$\left(ANN_{i}\right)$ represents actual effort of the particular project, which is calculated based on ANN-OA18, ANN-OA12, and ANN-OA16.

**Step 5:** For each iteration in our experiment, the output values are obtained according to Formulas (A5) and (A6):

(A5) $$MRE=\frac{1}{n}\overset{n}{\underset{1}{\sum }}\left|ActEfort-EstEfort\right|$$

(A6) MMRE=mean(MRE)

For each study part in each iteration, the gradient descent (*GA*) is monitored with the condition *GA* < 0.01, calculated as (A7):

$$GA=MRE_{i1}-MRE_{i2}<0.01$$

(A7) $$where\;i=\bar{1,n}\;n\;is\;number\;of\;ANN$$

The difference of the minimum values for each iteration in each *ANN architecture* is denoted by *delta (i)* = *δ_i_* and is calculated as follows (A8) and (A9):

(A8) $$\begin{array}{c} \delta_{i}=\left(OA\left(ANN_{k}\right)\;–\;OA\left(ANN_{k-1}\right)\right)Fm\;i-number\;of\;ANN,\;k-number\;of\;iteration,\;m\\ -number\;of\;risk\;factors \end{array}$$

(A9) $$\begin{array}{c} if\;\delta_{i}>\delta_{i+1}\;,\;then\;ANN_{i}\;converges\;to\;MMRE_{i}\;for\;each\;of\;the\;proposed\;ANN\;\\ architectures \end{array}$$

With this, in our experiment with different *ANN architectures*, we set the criterion of stopping convergence (number of iterations) at *GA* < 0.01. In the training phase of the candidate of the selected ANN architecture according to Taguchi’s orthogonal array, in each subsequent iteration, a reduction in MRE of less than 1% is achieved, which, in our experiment, represents a “stop criterion” [36].

**Step 6:** Examination of the influence of input values on the change of risk factors values:

1. The influence of the first input parameter and his value is calculated as (A10)–(A13): (ANN-OA12)

(A10) $$\;\delta 1=\;\mathrm{mean}\;\left(\mathrm{OA}\;\left(ANN_{k}\right)\right)–\mathrm{mean}\;(\mathrm{OA}\;\left(ANN_{k-1}\right)\;F1\;$$

where OA$\;\left(ANN_{k-1}\right)\;$F1 is mean (OA$\;\left(ANN_{k-1}\right)\;$F1 when X1 = 0; X1 = BMI;

2. The influence of the second input parameter and his value is calculated as:

(A11) $$\;\delta 2=\;\mathrm{mean}\;\left(\mathrm{OA}\;\left(ANN_{k}\right)\right)–\mathrm{mean}\;(\mathrm{OA}\;\left(ANN_{k-1}\right)\;F2\;$$

where OA$\;\left(ANN_{k-1}\right)\;$F2 is mean (OA$\;\left(ANN_{k-1}\right)\;$F2 when X2 = 0; X2 = Cholesterol;

3. The influence of the third input parameter and his value is calculated as:

(A12) $$\;\delta 3=\;\mathrm{mean}\;\left(\mathrm{OA}\;\left(ANN_{k}\right)\right)–\mathrm{mean}\;(\mathrm{OA}\;\left(ANN_{k-1}\right)\;F3\;$$

where OA$\;\left(ANN_{k-1}\right)\;$F3 is mean (OA$\;\left(ANN_{k-1}\right)\;$F3 when X3 = 0; X3 = Physical activity;

4. The influence of the fourth input parameter and his value is calculated as:

(A13) $$\;\delta 4=\;\mathrm{mean}\;\left(\mathrm{OA}\;\left(ANN_{k}\right)\right)–\mathrm{mean}\;(\mathrm{OA}\;\left(ANN_{k-1}\right)\;F4\;$$

where OA$\;\left(ANN_{k-1}\right)\;$F4 is mean (OA$\left(ANN_{k-1}\right)\;$F4 when X4 = 0; X4 = The family history; [36].

**Step 7:** The Pearson’s [35], Spearman’s [35], and R^2^ [37] coefficients are monitored.
